# Acknowledgment to Reviewers of *Diagnostics* in 2021

**DOI:** 10.3390/diagnostics12020484

**Published:** 2022-02-14

**Authors:** Diagnostics Editorial Office

**Affiliations:** MDPI AG, St. Alban-Anlage 66, 4052 Basel, Switzerland

Rigorous peer-reviews are the basis of high-quality academic publishing. Thanks to the great efforts of our reviewers, *Diagnostics* was able to maintain its standards for the high quality of its published papers. Thanks to the contribution of our reviewers, in 2021, the median time to first decision was 19 days and the median time to publication was 37 days. The editors would like to extend their gratitude and recognition to the following reviewers for their precious time and dedication, regardless of whether the papers they reviewed were finally published:
Aalap ChokshiJong Kook ParkAaron PollettJongho JeonAbdeldjalil OuahabiJonghyun ShinAbdelnaby KhalyfaJoo Beom EomAbdou ElhendyJoo Hyun OAbdul Rauf BaigJoon W. ShimAbdullah BayramJoost Van Der VorstAbedalrhman AlkhateebJordi RiberaAbhay ShahJörg MarotzAbheepsa MishraJorge Esquiche LéonAbhijit AithalJorge Hernández-BelloAbhilash PerisettiJorge M. Charco CharcoAbhilash VenugopalanJorge N.R. MartinsAbhishek Kumar SinghJorge RoaAbhishek SethJorge Santos-JuanesAbiodun Morakinyo AdeolaJorge TorresAbul BasharJosanne VassalloAchille TarsitanoJosé A. Jiménez HeffernanAda Maria FlorenaJosé A. Noguera-VelascoAdam GłowaczJose A. Regla-NavaAdam JanasJosé Angelito U. HardilloAdam PiorkowskiJosé Antonio HorcajadasAdam ReichJosé Antonio Morales-GonzálezAdam WylęgałaJose Antonio ParraAdelina JianuJose Antonio Vega AlvarezAdhikarimayum Lakhikumar SharmaJosé Bañuls-RocaAdina HutanuJosé Caparros-MartinAditya BansalJose Francisco Saenz-CogolloAdnan GhaffarJosé Javier Zamorano-LeonAdolfo PomaJosé López CastroAdrià ArboixJosé Luis Pardal-RefoyoAdrian BradyJosé Luís Sánchez-CarazoAdrian Elmi-TeranderJosé M Rodrigo-MuñozAdrian GoldisJosé Manuel CabedaAdriana Aguilar-LemarroyJosé Raduan JaberAdriana Herrera-GarcíaJosé Rafael De AlmeidaAdriano PiattelliJosé-Antonio Mirón-CaneloAdriano Polican CienaJosef BlazekAdriano TagliabracciJosef HalamekAdrienne K. SalmJosé-María Sánchez-GonzálezAdrienne L. GrzendaJosep Carles Benítez-MartínezAdrija HajraJoseph GeradtsAfroditi NanouJoseph MellorAgata GabryelskaJoseph P. HornakAgata MikolajczykJosephine K DermawanAgnes FleuryJosette RaymondAgnes KlarJoshua GawlitzaAgnese SbrolliniJosip A. BorovacAgneta SimionescuJosko BozicAgnieszka Gadomska-GajadhurJoško MarkićAgnieszka H. Ludwig-SłomczyńskaJoško OsredkarAgnieszka KordekJouha MinAgnieszka KosowskaJouke DijkstraAgnieszka Kulczyńska-PrzybikJoy AllenAgnieszka Lecka-AmbroziakJózsef PiffkóAgnieszka LemanskaJózsef PrechlAgnieszka LobodaJuan Bautista De SanctisAgnieszka MatuszewskaJuan C. De Agustin-AsensioAgnieszka MlynarskaJuan Camara SerranoAgostino GuidaJuan De Dios Berná-SernaAgostino SteffanJuan M. Mayor-TorresAgustin Enciso-MartinezJuan MelchorAharon WegnerJuan Mozas-MorenoAhmad R SedaghatJuan TorrasAhmad SayasnehJuei-Tang ChengAhmed Abd El WahedJuergen SchrezenmeirAhmed Abdel Khalek Abdel RazekJui-Ming LiuAhmet Muhteşem AğıldereJulia A. ScottAhsan HameedJulia GilhodesAida PetcaJulian S. PetersAida PitarchJulie Brogaard LarsenAikaterini TerzoudiJulie MagatAïmen KhacharemJulien DemiselleAishwarya IyerJulija ArmalyteAjay KevatJung-Soo PyoAjeet KaushikJun-Jen LiuAkihiko MiyanagaJun-Jun YehAkiko HanyudaJunkichi YokoyamaAkinari SawadaJun-O JinAkira KitamuraJunseok ParkAkira KurataJunxuan MaAkshara RaghavendraJun-Young KimAkshata R. NaikJürgen GermannAkshay PandeyJürgen PannekAlarico ArianiJustin VranicAlbano DomenicoJustyna MiszczykAlberto AntonelliJustyna SzumiłoAlberto BalduzziJu-Yi ChenAlberto BollettaJyotir ChatterjeeAlberto BongiovanniJyotsna ShahAlberto CaggiatiK. H. Katie ChanAlberto CiprianiK. KisandAlberto Enrico MaraoloKa Fung Peter ChiuAlberto Herreros LópezKa L. HongAlberto Lo GulloKadhim KadhimAlberto MellaKai ZhangAlberto MiceliKailash KrishnanAlberto Rodríguez-ArchillaKalevi KairemoAlberto SignoreKalpana RajaAlbino EccherKamal JoshiAldona KowalskaKamal Kant SahuAleix SolanesKamil GrubczakAlejandro Galán-MercantKamil KrawczynskiAlejandro Martínez-ÁguilaKamila GoderskaAlejandro Pérez-ÉcijaKamran ShaukatAlejandro TelloKana FujikuraAleksandar VišnjićKandasamy SaravanakumarAleksander F SikorskiKannie ChanAleksander Jerzy OwczarekKaoruko ShimizuAleksandr A. RubelKarel D. CapekAleksandr RakhmangulovKarel KotaškaAleksandra Karczmarska-WódzkaKarel RoubikAleksandra KrolikowskaKaren Cowden DahlAleksandra PfeiferKaren TanAleksandra ŻebrowskaKarim RehmanAleksey Michailovich ChaulinKarin Åström OlssonAlenka Repše FokterKarin Markenroth BlochAlessandra AlteriKarin NylanderAlessandra BorgheresiKarl BechterAlessandra FiorettiKarl Reinhard AignerAlessandra LaforgiaKarol Rawicz-PruszyńskiAlessandra PullieroKarolina PierzynowskaAlessandra StasiKaryna IsaievaAlessandro ArenaKatalin MikeczAlessandro CapucciKatalin PetőAlessandro CarrerKatarina VukojevicAlessandro CarrieroKatarzyna Borycka-KiciakAlessandro CeliKatarzyna Kościelska-KasprzakAlessandro De SireKatarzyna KrysikAlessandro De VitaKatarzyna ŁukasiukAlessandro FancelluKatarzyna Mizia-StecAlessandro FantiKatarzyna NowomiejskaAlessandro FratiKaterina RoubalovaAlessandro IeraciKatharina HessAlessandro MalobertiKatherine ElferAlessandro ManconKathleen (Katie) DorrisAlessandro MangognaKathryn Y. BurgeAlessandro MeduriKatia BarbaroAlessandro PerrellaKatia StefanantoniAlessandro PiniKatie F. LovesonAlessandro PosaKatie MeehanAlessandro RizzoKatja VetvikAlessandro RussoKatrin SchierleAlessandro SteccoKatsuaki IeguchiAlessandro StefanoKatsuhiko IshiharaAlessia CimadamoreKatsuhiro ItoAlessia PellerinoKaushik SridharAlessia SaricaKavita SharmaAlessia SpadaKavitha SunasseeAlessio ArdizzoneKay Choong SeeAlessio RossiKay Dietrich WagnerAlex Wing Hong ChinKay WagnerAlexander FilatovKazimierz KarolewskiAlexander H. MaassKazuhiko KibayashiAlexander HartmannKazuhito SuzukiAlexander HeinleinKazuki IdeAlexander KayKazumichi FujiokaAlexander PemovKazuo KubotoAlexander PisarchikKazuya AkahoshiAlexander RobitzschKazuya MimuraAlexander SweetmanKazuyoshi IshidaAlexander TarrK.C. SantoshAlexander V. ProninKe WangAlexander WreeKee-lung ChangAlexandr KokovKeigo MurakamiAlexandra MalinovskaKeiichi HironoAlexandra NunesKeiko UedaAlexandra Ripszky TotanKeisuke MurakamiAlexandre G. De BrevernKeita HiraiAlexandre KauskotKelechi NjokuAlexandros DaponteKelly DomvriAlexandros KarakostisKelvin WongAlexandru BurlacuKen MuramatsuAlexandru PopaKen TakasawaAlexey BoykoKenichi SudaAlexey ChubarovKenji OhbaAlfonso MastropietroKenjiro YamamotoAlfred O. AnkrahKenneth HettieAlfredo CaturanoKensuke MitsunariAlfredo Di LeoKentaro TojoAlfredo G. CasanovaKentaro YamaoAlfredo R. GalassiKentaro YoshiokaAlfredo VellidoKenya KusunoseAli AkalinKeun-Yeong JeongAli Al KaissiKevin BouillerAli FarahaniKe-Vin ChangAli Hussein EidKevin Chien-Chang WuAli MobasheriKevin FonsecaAli Mohammad AlqudahKevin RolfeAli Salehzadeh-YazdiKhaled ElsayadAli SelamatKhayriyyah Mohd HanafiahAlice BoilèveKhin ThaAlice Georgia VassiliouKi Jin JungAlice M TurnerKieran HalloranAlice RichardsonKifayat UllahAlicia Rodriguez-PlaKihwan ChoiAlida VelooKilian WeigandAlina VedutaKimberly H. WoodAlphus D WilsonKimberly PerezAlpo VuorioKinan AlhallakAltaira D. DearbornKinga Grzech-LeśniakÁlvaro Francisco Lopes De SousaKirill AntonetsAmadeus DobrescuKirsten LimesandAmanda IglesiasKirsten Van LangeveldeAmanda LosiKishan Kumar NyatiAmani Yousef OwdaKishore KumarAmarish YadavKishore Raj KumarAmbarish P. BhatKishu RanjanAmbra BuschiazzoKisoo PahkAmbra Del GrossoKi-Sun LeeAmelia Maria GamanKit WongAmelia PietropaoloKiyong NaAmelina AlbornozKiyoshi MasudaAmer ZakariaKlaus GratzAmerigo GiudiceKnut Tore-LappegardAmerigo VitaglianoKoen Van De VijverAmeya D. JagtapKoichi KitagawaAmgad HannaKoichi MatsuoAmir R. KachooeiKoji FujitaAmir SohrabiKoji KanayamaAmira MoawadKoji MitaAmirmasoud AhmadiKoji OtaniAmirreza MahbodKondapa Naidu BobbaAmit KumarKonstantin P. GaikovichAmna ParveenKonstantina FotouAmoako DanielKonstantinia AlmpaniAna BlascoKonstantinos A. GatzoulisAna BoroveckiKonstantinos A. ZikopoulosAna CandiotaKonstantinos KatogiannisAna CaruntuKonstantinos MakrisAna Cristina BragaKonstantinos MisiakosAna Cristina GonçalvesKonstantinos SapalidisAna CrnkovicKonstantinos StergiosAna CusumanoKonstantinos TosiosAna Elena Pérez-CobasKostas PerisinakisAna Elvira Rodríguez-SotoKota TsubouchiAna FaustinoKota YokoyamaAna Gonzalez-MarcosKoumantakis GeorgeAna GradissimoKourosh SheibaniAna Isabel PlácidoKrishan RaiAna Luisa González-Celis RangelKrishna HortAna Margarida AbrantesKrishna KantAna María García-VicenteKrishna Mohan SurapaneniAna R. OchagavíaKristen M. MeiburgerAna Rita GomesKristen M. TecsonAnabela Baptista Pereira PaulaKristijan SkokAnamarija ŠestakKristína CzekóováAnand ChandrasekharKristina KuhbandnerAnand Prakash SinghKristóf Endre KárolyAnastasia DesyatovaKristoffer Lindskov HansenAnastasia HyrinaKrystallenia AlexandrakiAnastasia KotanidouKrzysztof GorynskiAnastasia MitseaKrzysztof KaliszewskiAnastasios DoulamisKrzysztof KrystaAnastasios MarkopoulosKrzysztof LetachowiczAnat O. Stemmer-RachamimovKrzysztof ŚlosarekAnatol PanasiukKseniya RubinaAnca AvramKuangyu ShiAnca ColitaKuldeep AttriAnca DoceaKumánovics GáborAnca PanaitescuKumi SmithAnca SimionescuKunhua SongAndaleb KholmukhamedovKunito OkuyamaAnders BergqvistKuo-Chuan HungAnders HjerpeKuofeng HungAnders JacobsenKuo-Hu ChenAnders KaestnerKyeongryoon LeeAndrás BánvölgyiKyle T. AmberAndras BikovKym LowryAndrás BikovKyong IhnAndraz DovnikKyung Eun LeeAndré Luis Costa-Da-SilvaKyung-Yil LeeAndre WirriesLaetitia VercellinoAndrea AlexandreLaffranchi MattiaAndrea AngelettiLaia Rodriguez-RevengaAndrea AngeliniLai-San WongAndrea BaragettiLaith AlzubaidiAndrea BarbieriLakshmanakumar KinthadaAndrea BeccioliniLakshmi PrabhuAndrea BorghesiLal HussainAndrea BrugnoloLamia Ait-AliAndrea BuronLan MiAndrea Casas-VargasLara SchlaffkeAndrea CordaLarisa AnghelAndrea CozziLars C. GormsenAndrea FrasoldatiLars T JensenAndrea Gonzalez-MontoroLászló CzakóAndrea MartiniLaura Anna LeoAndrea Paola Rojas GilLaura AudìAndrea RinaldiLaura BergantiniAndrea SambriLaura BurattiniAndrea SzékelyLaura CaponiAndrea TangherloniLaura CominiAndrea VergalloLaura CotoiAndrea ZbindenLaura EvangelistaAndreas EbneterLaura Francés-SorianoAndreas GaumannLaura KasmanAndreas SchmidLaura M. Benítez-GutiérrezAndreas SeitzLaura MaziluAndreas Von KnethenLaura Mora-LópezAndreas-Antonios RoussakisLaura PelizzariAndreea RatiuLaura PeraltaAndreia L. PintoLaurel O. SillerudAndreina BajLaureline BerthelotAndrej ThurzoLaurence CarrollAndreja Trojner-BregarLaurent BélecAndres Iglesias PrietoLaurent MetzingerAndrew BentleyLaurent MullerAndrew CollierLaurent SoustelleAndrew DonsonLazar B. DavidovicAndrew G. MarshallLazzaro Di BiaseAndrew LaneLee Samüel WannAndrew MacnabLei WanAndrey ChernovLeo A. KimAndrey LvovLeonardo BascoAndrey SimbirtsevLeonardo GabrielliAndrii SlonchakLeonardo Jose Mataruna-Dos-SantosAndrzej GlowniakLeonardo Juan Ramirez LopezAndrzej GrzybowskiLeonardo OggianoAndrzej KoniecznyLeonardo RundoAndrzej MaterkaLeszek SzalewskiAndrzej PolańczykLetao YangAndy Wai Kan YeungLeticia Bernal-MartínezAneta Cymbaluk-PloskaLeticia Martín-CorderoAneta Ostróżka-CieślikLeyre ZubiriAngela CaponnettoLi HeAngela CastoldiLi LiAngela DieterichLi Linda.TAngela MastronuzziLi Yueh HsuAngela SantoroLiana Figueiredo NobreAngela SpanuLiang KangAngeliki AngelidiLi-Ang LeeAngelo CarrettaLiang LiangAngelo PorrecaLiang-Yu ChenAngelo SiricoLianhua DongAngelo ZulloLicun WuAngelos SikalidisLidia GattoAngie FasoulaLilach SoreqAnida Maria BabtanLiliana ChemelloAniela PopescuLiliana DumitracheAniello MaieseLiliana Szyszka-SommerfeldAnil Kumar JonnalagaddaLin Ching ChangAnimesh AcharjeeLin LiAniruddha RayLina A.S. CarvalhoAnja C. RodenLina GumbienėAnjan GudigarLina YoussefAnjul KhadriaLinbo ZhaoAnkana KakotiLinda M. CarrollAnkit GuptaLingeng LuAnkit ManglaLin-Li ChangAnkur SainiLiqun JiaoAnn A. VerhaegenLirong WangAnn IgoeLisa DannenbergAnna Caterina MilanettoLiselle BovellAnna Chachaj-BrekieszLiselotte HardyAnna Erkiert-PolgujLivia PuljakAnna Kablak-ZiembickaLiviu-Adrian CotfasAnna Lena FisseLi-Yueh HsuAnna Maria HeikkinenLjubisa TopisirovicAnna MertasLobo LouieAnna MosiołekLong JinAnna N. BerlinaLongo NicolaAnna NowinskaLoredan NiculescuAnna PalmisanoLoredana BellantuonoAnna Paradowska-StolarzLoredana MarcuAnna PasettoLoredana Sabina Cornelia ManolescuAnna PołećLorena IncorvaiaAnna S. TzortziLorenz LeitnerAnna SobiepanekLorenz Lorenz RischAnna StarzyńskaLorenzo BonomoAnna SynakiewiczLorenzo FaggioniAnna WardowskaLorenzo GenitoriAnna WaśniewskaLorenzo MannelliAnnafranca CavaliereLorenzo ScappaticcioAnnalisa PaceLorenzo UggaAnnalisa SantucciLoris NanniAnnamaria NicolliLorraine Z. MutsvungumaAnnarita FanizziLory Saveria CrocèAnnarosa FloreaniLouis J. VaickusAnne L. PiantadosiLouis LassalleAnne Marie Kuijpers-JagtmanLouisa BolmAnne PohlmannLuana Andreea MacoveiAnne Vest SørensenLubica RauovaAnnelieke E.C.A.B. WillemsenLubomir SkladanyAnnette AudigéLuc BidautAnnibale VersariLuca AmbrosioAnnie VahedipourLuca ArdigòAnnika AuranenLuca BoeriAnnovazzi AlessioLuca CamoniAnoop EnjetiLuca ChiovatoAntal NógrádiLuca Di GianfrancescoAntesar M. ShabutLuca FalzoneAnthony DobiLuca FilippiAnthony GloverLuca GiacomelliAnthoula GaigneauxLuca GiannellaAntimo CutoneLuca Giovanni LocatelloAnton FaronLuca MagistrelliAnton PetrovLuca MarinelliAntonia Dimitrakopoulou-StraussLuca MenichettiAntonia KaltsatouLuca MoroniAntonia RibesLuca NicosiaAntonino Lo GiudiceLuca PaciniAntonio BarileLuca PaduaAntonio BerizziLuca PierelliAntonio BiondiLuca ProiettiAntonio BulumLuca RevelliAntonio CentofantiLuca SteardoAntonio CP WongLucchese AlbertaAntonio D’AmatiLucia A. SealeAntonio FacciorussoLucia AnsaniAntonio FortunatoLucia BlascoAntonio GiordanoLucía FernándezAntonio GiorgioLucia FinocchioAntonio GranataLucia LeccisottiAntonio LucacchiniLucia NappiAntonio MaccioLucia StanciakovaAntonio ManentiLucia ZanoniAntonio Mario ScanuLucian BeerAntónio Miguel MorgadoLucian GruionuAntonio Palazón-BruLuciano MaestriAntonio ParzialeLucilla ParnettiAntonio PezoneLucio MontanaroAntonio PontorieroLucio TremolizzoAntonio RaffoneLucy MorganAntonio RapacciuoloLudmila Danilowicz-SzymanowiczAntonio SarolicLudovic Loup LhermitteAntonio ScaranoLudwig WagnerAntonio SchiattarellaLuigi Angelo VairaAntonio Simone LaganàLuigi BennardoAntonio ViúdezLuigi CarboneAntonis SakellariosLuigi CipolloniAnwar UsmanLuigi FortunaApostolos BeloukasLuigi LoscoAraceli García-MartínezLuigi MinafraAraceli Ortiz RubioLuigi P BadanoArash NavranLuigi PiazzaArchontis GiannakidisLuigi VetrugnoArgyro MaziotiLuigia D. De FalcoAria NouriLuigia De FalcoAriel KorenLuis ÁguilaArinc OzturkLuis Mariano EstebanAris AntsaklisLuis MonteiroAris TsalouchosLuis PianciolaArjan VissinkLuís Pinto Da SilvaArlène C. ChuaLuis Tallón-AguilarArmand AbergelLuisa María BotellaArmando CavalloLuisa PetroneArmando CocciaLuisa PieroniArnaldo StanzioneLuisa Warchavchik HugerthArnaud LombardLuis-Alfonso Arráez-AybarArnaud W ThilleLuise HolzhauserArrigo CiceroLuka Alexander ClarkeArtur CieslewiczLukas LacinaArtur RydoszŁukasz DembińskiArtur Tadeusz KrzyżakLukasz KrzychArturo BonomettiŁukasz ŁapajArunava GhoshŁukasz MałekArun-Kumar Kaliya-PerumalŁukasz MazurkiewiczAshis MondalŁukasz PaczesnyAshish J. MathewŁukasz PałkaAshkan Ghanbarzadeh-DagheyanŁukasz SzycAshok PatowaryLuke PaterAshraf M. MohieldinLuminita MoraruAshu JohriLuminiţa MoraruAshwanth Christopher FrancisLuminita PaduraruAsmerom SengalLuna GarganiAtashi SharmaLutgarde LynenAtef HobinyLydia Gimenez LlortAthanasios AnagnostisLydia Min-Ying SuAthanasios TragiannidisLynette HodgesAthikhun SuwannakhanLynne BingleAthina MarkouLyubov E. SalnikovaAtif ShahzadM. CellinaAtsuhiko YagishitaM IbragimovaAtsushi IrisawaM. Leontien Van Der BentAtsushi KannoM. Rashed KhanAtsushi MasamuneMaciej DabrowskiAtsushi TakaiMaciej KochanowskiAttila FrigyMaciej SkotakAttila KovariMaciej WalędziakAttila RaczMaciej ZielińskiAttila RokaMadelon C VonkAugust WrotekMads Radmer JensenAvinash AujayebMagda RybickaAviram HochstadtMagdalena Kocot-KępskaAvital Y. O’GlasserMagdalena Kowalewicz-KulbatAxel HartkeMagdalena OlbrytAxel RegeniterMagdalena RudzińskaAxel SteigerMagnus Bjorkavoll-BergsethAyako EdahiroMagnus FalkAyataka FujimotoMahdieh MoghisehAydin EresenMahesh KaushikAysegul AksanMahima SinghAyumi SugiuraMahmoud ElbattahAyyanar SivananthamMahsa DabaghAzam YazdaniMaik HaentschelAziz AmineMaik HerbigAzumi IshizakiMaja MatulićAzzurra StefanucciMakito MiyakeBabak SabouryMakoto AdachiBabu Pappu MohanMakoto EndoBahi TakkoucheMakoto HasegawaBahriye AktasMakoto NogamiBaicus AndaMałgorzata GrabarczykBailin LiuMalgorzata GrzesiakBalaji PanchapakesanMalgorzata MaciukiewiczBalazs MargitMałgorzata Natalia MojsakBálint NagyMałgorzata PrzybyłoBandar AlosaimiMalgorzata RusakBang-Bin ChenMałgorzata StefańskaBarbara BrognaMalte KircherBarbara J. FuegerManabu NatsumedaBarbara JarząbManal FawzyBarbara MiziakManel Ben HammoudaBarbara PalumboManfred WeidmannBarbara RuaroManfred WickBarbara Ruszkowska-CiastekM.Angela PascualBarbara Sosnowska-PasiarskaManh Cuong HoangBarbara Y. WhitmanMani AlikhaniBardia YousefiManisha WitmansBartlomiej GorskiManjung HungBartłomiej Henryk NoszczykMansi SharmaBartłomiej NiespodzińskiManuel Aguilar-DiosdadoBartłomiej PerekManuel De RojasBartosz MałkiewiczManuel MataBartosz PrzysuchaManuel RequenaBartosz WojciukManuel ScimecaBayu Adhi TamaManuel VilelaBeata JabłońskaManuel WeberBeata KaletaManuela ColucciBeata Kasztelan-SzczerbińskaManuela LudovisiBeata Sarecka-HujarManuela MatesanBeata Uziębło-ŻyczkowskaMaolin LuBeatrice LeonardiMar DomingoBehnam RashidiehMara MarongiuBehzad Behzad FarahaniMarc BarritaultBelal AlsinglawiMarc FischlerBen CostelloMarc HilhorstBenedetto LongoMarc LütgehetmannBeniamin Oskar GrabarekMarc SaakeBenjamin LampMarcello CiaccioBenjamin LongereMarcello DonatiBenjamin MetcalfeMarcello RomanoBenjamin SassMarcelo Sousa SilvaBenjamin UlrichMárcia Antoniazi MichelinBeomseok KoMárcia BarrosBernard DucheneMarcin KurzynaBernardo VegaMarcin OpławskiBernd SchweizerMarcin RozalskiBernd StratmannMarcin SamiecBernhard PetritschMarcin WełnickiBernhard ZelgerMarcin WłodarczykBernward LauerMarco AllinoviBerthold HocherMarco ArmbrusterBertrand MathonMarco BattistaBetrouni NacimMarco CasteleijnBettina BlaumeiserMarco DelsanteBettina HeiseMarco FerrariBharath Babu NunnaMarco InvernizziBharath RamaswamyMarco IosaBhasker RadaramMarco ManfrediniBhola PradhanMarco MascittiBhola Shankar PradhanMarco Matucci CerinicBhopal MohapatraMarco MoschettaBianca BesteherMarco PaciottiBianca GalateanuMarco PezziBianca NitzscheMarco PicardiBianka ForgoMarco PuthenparampilBikbov Mukharram M.Marco SperandeoBin SongMarco TjioeBingqian QuMarcos AndreBiraja GhoshalMarcos Lõpez-HoyosBirgit HoferMarcus GrimmBjarke JensenMarcus HorstmannBlanca De-la-Cruz-TorresMarek DedecjusBo Kyung ChaMarek WesołowskiBoban M. EROVICMarek WideraBogdan Silviu UngureanuMargaret BublitzBogdan SoceaMargaret J. LangeBoguslawa LuzakMargarita A. SazonovaBonnie WebsterMargarita KirienkoBon-Sang KooMari YuasaBoohwi HongMaria A. BaturovaBorek SehnalMaria Angela LosiBoris A. YakobsonMaría Ángeles De MiquelBožena Ćurko-CofekMaria Antonia FrassanitoBrad BarnettMaria Assunta ZoccoBrajesh Kumar SinghMaria Chiara AmbrosettiBrandon G. SmagloMaria Colomba ComesBrandon Lucke-WoldMaria ContaldoBratko FilipičMaria Cristina CuriaBrendan C. StackMaria Cristina GauzziBrendan C. Stack, Jr.Maria D. Tomai PitincaBrendan J. HealyMaria De BonisBreznik BarbaraMaría De-la-Casa-AlmeidaBrian BarthMaria Elena LainoBrian D. AdamsMaria Elena RiccioniBridget F. KoontzMaria FortofoiuBrigid Betz-StableinMaria G. KoutelouBrigitte HantuschMaria Grazia MancinoBrigitte WilczekMaria Grazia MatarazzoBritt-Isabelle BergMaria Grazia PiancinoBrock HumphriesMaría Hernández-SánchezBrock JensenMaría José BuitragoBruno FiondaMaría José García-PolaBurkhard RiemannMaria K. SvenssonByung Soo KimMaria KourtiC. Sieiro SantosMaria KyritsiCaius ConstantinescuMaria LeeCălin CăinapMaria LucaCălin-Adrian PopaMaria Luiza S. MelloCalogero CasàMaría M. Morales Suárez-VarelaCamelia Sorina StancuMaria Marcella LaganaCamila D. Madeira CamposMaria MilettaCamilo G. SotomayorMaria MojicaCarina MalmhällMaria MonsalveCarla LoretoMaria MorrisseyCarla Solé CañadasMaria MrówczyńskaCarlo Alberto RicciardiMaria PiancinoCarlo AprileMaria PicchioCarlo Augusto MallioMaría Pilar Pecci-LloretCarlo FabbriMaria SalsoneCarlo GaniniMaria ScaturroCarlo LaviaMaria Teresa RocchettiCarlo PapponeMaria Teresa VietriCarlo SaccardiMaria TotanCarlos G. JuanMaria TrovatoCarlos Maria GalmariniMaria Vittoria CubellisCarlos Martin LlorenteMaria-Alexandra MartuCarlos Navarro CuéllarMaria-Alexandra PaunCarlos SilvaMaria-Fernanda Lorenzo-GómezCarmel StockMariagabriella PuglieseCarmela PaolilloMariagrazia PiccioniCarmelina C. ZirafaMariana BentoCarmelo CaldarellaMariana Cardoso DiogoCarmelo MilitelloMariana TudoranCarmen CadillaMarianna ArvanitakiCarmen CamaraMarianna DelussiCarmen De MendozaMarianna RanieriCarmen Méndez-HernándezMarianne A. JakobsenCarmen RinaldiMariano Amo-SalasCarmenza SpadaforaMariano RocchiCarmine FinelliMariano Sánchez CrespoCarol StanciuMariarita BrancaccioCarol Yen Chin LinMaribel LopezCaroline BeardsmoreMarie France BellinCătălin Ioan VladMarie LubyCatalin StoeanMarie-Pierre GolinelliCatalin TiliscanMarija HefferCatarina GodinhoMarika TardellaCaterina GiannittoMarilena MinieriCecile LepageMarina HodolicCecilia BindaMarina MuzzaCecilia EstradaMarina OteleaCemil ColakMarina ZajnulinaCésar Calvo-LoboMarinella CocoCesar Díaz-TornéMarino Menozzi JäckliCesar Leal CostaMario CioceChaenyung ChaMario CrucianiChaido SirinianMario D’ AcuntoChaithanya ChelakkotMario GruppoChan Kuan RongMario LimaChanchal MandalMario MascalchiChang Dong YeoMario VersaciChangho LeeMarion RyanChang-Hoon ChoiMarisel Romina TuttobeneChang-Sei KimMariusz KusztalChang-Shen LinMark A. ChilversChanpreet SinghMark A. GreenChantal DysliMark BustorosChao WangMark GriffthsChara PapadakiMark K. FarrugiaCharalampos LoutradisMark McGurkCharalampos ThomasMark S. ScherCharanya KumarMark Van GastelCharat ThongprayoonMark ZaninCharilaos KoulourisMarkus BlaessCharles D. SearlesMarkus HoopmannCharles MarksMarkus J. AnkenbrandCharles MesguichMarkus KrebsCharles TruilletMarta GrecoCharles VerneyMarta MazurCharlotte BeaudartMarta OpalińskaCharu KothariMarta VerneroChe-Hung ShenMarta ZerunianChengheng LiaoMartin ChanCheng-Hsien LiuMartin KeuchelCheng-Hsun HoMartin KoestenbergerCheng-Maw HoMartin LoertscherCheng-Yen KaoMartin MuellerChen-Hua LiuMartin P. PlayfordChenyu SunMartin ProescholdtCheol E. HanMartin SeipenbuschChester J. JoynerMartin SpitzerChi Chun WongMartin ZiegerChia-Hwa LeeMartina BonifaziChiao-En WuMartina ChiriacòChiao-Ling TsaiMartina DermeChiara AcanforaMartina FerioliChiara CarlomagnoMartina MeszarosChiara CastellaniMartina PiccoliChiara CortiMartti VastamäkiChiara Maria GranaMartynas TalaikisChiara MarracciniMarvin Coto-JiménezChiara MartiniMary N. ShephardChiara PiubelliMaryam ArdalanChiara RossoMarzena BieleckaChiara TurchiMarzia BedoniChiara VillaMarzia SeguChichun HoMarziliano NicolaChien-Chin ChenMasaaki KomatsuChien-Feng LiMasaaki KonishiChien-Ming ChaoMasaaki ShimataniChih-Che LinMasahiko InamoriChih-Jen YangMasahiro TanabeChih-Yang HsiaoMasahito AsadaChihyang WangMasakazu FujimotoChi-Ju KimMasamichi ISHIAIChing-Yao YangMasanobu IbarakiChin-Mu HsuMasaru TanakaChintalapudi NaliniMasataka KikuyamaChiranjit GhoshMasato AragakiCho-Hao LeeMasato YasumotoChris BoydMasaya AkashiChrissa SiokaMasayuki NagasawaChristian F. FreyschlagMassimiliano De PaolisChristian HappelMassimiliano EspositoChristian JenssenMassimiliano MangoneChristian MorgensternMassimiliano SorbelloChristina AndicaMassimiliano ValerianiChristina MejersjöMassimo GalliChristine Attenhofer JostMassimo GeunaChristine DewiMassimo IacovielloChristine PratilasMassimo LibraChristoph F. DietrichMassimo MaccagnoChristoph JochumMassimo MarrelliChristoph M. FriedrichMassimo MattioliChristoph MitschMassimo MilioneChristoph R. BehemMassimo SalviChristoph ThomssenMatea Nikolac PerkovicChristophe PadoinMatei BratuChristopher KobierzyckiMateusz Jacek SpałekChristopher MontemagnoMateusz KwitniewskiChristopher PayanMateusz MaciejczykChristopher S GondiMateusz WiniarczykChristos ChatzikyrkouMathieu GautierChristos K. KontosMathieu JozwiakChristos MikropoulosMathieu QuinodozChristos SachpekidisMatías MorínChristos TsalikidisMats NilssonChuansheng ZhengMatteo BadalamentiChueh-Hung WuMatteo BaucknehtChuen-Fu LinMatteo BolcatoChul-Ho OakMatteo Bruno LodiChu-Lin TsaiMatteo ChiappediChun WangMatteo CioccaChun-An ChengMatteo InnocentiChun-Chih TsengMatteo NeriChunfu YangMatteo RicchiChung Y. HsuMatteo RiccòChung-Feng LiuMatteo SchimberniChung-Jung LiuMatteo SuterChung-Lieh HungMatteucci FedericaChun-Hou WangMatthew A. VaracalloChun-Ru ChienMatthew J. ThompsonChunyi WenMatthew PetersonChun-Yu ChenMatthew R. BurasChuyi ChenMatthias EberhardCinzia CaliendoMatthias F. FroelichCinzia GiacomettiMatthias FröhlichCinzia MasperoMatthias GaidaCinzia MortarinoMatthias GrotheCiprian JurcuţMatthias GüntherCiprian RezusMatthias KarlCiprian TomuleasaMatthias NeesClaire JosseMatthias P. FabritiusClaire PearsonMatthias PriemelClaire Rodriguez-LafrasseMatthieu VignesClara HjalmarssonMattia Di PaoloClarence KhooMattia DominoniClaudia Angela Di SilvestriMattia GentileClaudia CavaMattis GeigerClaudia MehedintuMaura MiccoClaudia SommerMaurizio AcampaClaudia WeidensteinerMaurizio EliaClaudio GambardellaMaurizio PocchiariClaudio PasqualiMaurizio RicciClaudio TanaMaurizio ScarpaClaudio UcciferriMaurizio TonettiClaus Bo SvendsenMauro GiuffrèClaus PetersenMauro Giuseppe MastropasquaClaus Vinter Bødker HviidMauro MontalbanoClementina López-MedinaMauro RagoneseCleo-Aron WeisMawieh A HamadCodruta StoicaMax ZimmermannColette Kanellopoulos-LangevinMay BisharatColleen M. McDowellMazin Abed MohammedColleen SteinMd Mostafa Kamal SarkerColm O’RourkeMehmet Emin AdinColt EgelstonMehmet M AltintasConceição LoboMei-Huei ChenConcetta Di NoraMeiji Soe AungCongshan SunMeiqing ShiConsolato M. SergiMelanie BarzConsolato SergiMelanie FoeckingConstantina AggeliMelissa BorrelliConstantino Carlos Reyes-AldasoroMelissa EdwardsConstantinos BakogiannisMelissa PreissnerCorina PienarMenelaos ZafrakasCorina S. PopMengyu WangCorinna GrasemannMercedes Casanova MitjavilaCornelia AmalineiMi Suk JeongCornelia BraicuMichael AnapolskiCorneliu MunteanuMichael AradCorrado AngeliniMichael CalcuttCorrado CampochiaroMichael ChristCorrado PaganelliMichael D. GrayCorrado RomanoMichael D. SpartalisCorrado TagliatiMichael DöllingerCosimo BruniMichael E. HylandCosimo CumboMichael EichbaumCosimo NardiMichael HummelCosimo SpertiMichaël LaurentCostantino CaroselliMichael LiebmanCostantino CiallellaMichael OnkenCraig ErkerMichael P WillandCristian Castillo OleaMichael PretterklieberCristian FioriMichael R. GoCristian Iulius SurcelMichael RoseCristian NavarreteMichael SchreiberCristian StătescuMichael T. KoltzCristiana Iulia DumitrescuMichael VannierCristiano AmarelliMichail E. KlontzasCristiano CarbonelliMichail KalogiannakisCristina BorziMichał Jan StasiowskiCristina CapatinaMichał LatalskiCristina CasalouMichal LewandowskiCristina ChircovMichal LinialCristina CiucaMichał P. WasilewiczCristina FerrariMichał PomorskiCristina Galache OsunaMichal S. NowakCristina MuellerMichał StrzeleckiCristina PopMichal SzeremetaCristina Roldán-JiménezMichał-Goran StanišićCristina RuedaMichalis PanteliCristina RusuMichel MeignanCristoforo PomaraMichela GabelloniCrystal M. GiganteMichela IannoneCyprian OlchowyMichela PoliciCytology José A. Jiménez-HeffernanMichela RossiD Gareth EvansMichèle CaragliaD. Gareth EvansMichele D’AttilioD.A. CrosbyMichele Di CosolaDace BerzinaMichele DicataldoDae-Hyun JangMichele FusaroDae-seok HwangMichele GhidiniDafna SussmanMichele LanzaDafne BongiornoMichèle M. KindDagmar HorákováMichele MarchioniDagmar PavlůMichele MazzolaDagoberto Castellanos-NievesMichele OkunDaiji KawanamiMichelle MonaskyDaiji NagayamaMichiko NagamineDaisuke KikuchiMiglė BacevičienėDaisuke UtsunomiyaMiguel A OrtegaDale O. KiesewetterMiguel A. OrtegaDalia KaisarlyMiguel Ángel García IzquierdoDalius JatuzisMiguel Angel Sanchez-TenaDaly AvendanoMiguel Armengot-CarcellerDamian HudziakMiguel IdoateDamian KaweckiMiguel Lopez-FerberDamian LabudaMiguel Mascarenhas SaraivaDamiani GiovanniMiha SkvarčDamiano CaputoMihaela TurtoiDamiano CarusoMihai Emil CăpîlnaDamien HayeMihai Stefan MuresanDan VoiculescuMihaiela Cornea-CipciganDana BadauMihail Lucian BirsaDana HuskensMihalj BakatorDana LawrenceMihnea-Alexandru GamanDana Maria CopoloviciMiihir Narayan MohantyDana PopMika MononenDaniel A. MorrisMika SalmiDaniel B. YaroshMikel AldabaDaniel Dalla TorreMikhail MilchenkoDaniel EyraudMikhail ZubkovDaniel FriedMiki KushimaDaniel GeraghtyMilad MoloudizargariDaniel HerrMilad SalemDaniel P. PotaczekMilagros Hidalgo De La CruzDaniel PinkMilan PrsaDaniel PredaMilan TomaDaniel R. McGowanMilda PleckaityteDaniel RaffertyMilena AwadDaniel W. NixonMilena BellinDaniela AmzarMilena GeorgievaDaniela BoccoliniMilos PetrikDaniela CabibiMimi KimDaniela Di VenereMing ChenDaniela GasparottoMing Feng LiaoDaniela MessineoMing HuangDaniela Oprea-LagerMing Jie LeeDaniela Sonja NoschMing ZhangDaniela TalhadaMing-Ching HsiehDaniele ArmocidaMingchuan ZhouDaniele CastellaniMingchung ChouDaniele Della LattaMing-Hui SunDaniele DiacintiMing-Shao TsaiDaniele GarcovichMing-Tse KuoDaniele La ForgiaMinliang LiuDaniele MasaroneMinoru MiyazatoDaniele RiminiMinu PilvankarDanielle Aparecida GuimarãesMiodrag FilipovicDaniel-Mihai RusuMiquel Armengot-CarbóDaniil N. BratashovMira M. MilasDanijela Vrdoljak-MozetičMiran SkrapDanilo BuonsensoMircea-Catalin FortofoiuDaphne VassiliouMireia MallandrichDario Di NardoMireille B. KalouDario RahelićMiriam-Rose MenezesDario RuscianoMiriana D’AlessandroDario SiniscalcoMirko De MelisDariusz NowakowskiMirko ManettiDaša StupicaMiro JukićDasantila Golemi-KotraMitchell R. McGillDavid Ahmedt-AristizabalMitrea Delia-AlexandrinaDavid AllenMitsuaki IshidaDavid B. BeckMitsuru NakazawaDavid BirchMizuho NishioDavid J. CantyMobeen Ur RehmanDavid J PickettsMohamad AlamehDavid KalfertMohamad AssiDavid L. MayorMohamed ElsharkawyDavid MincherMohamed M. DesoukiDavid PérolMohamed MahdiDavid ReynosoMohammad AlamDavid RomascanoMohammad Ali SanjariDavid SloukaMohammad AzadDavid WoodMohammad Faraz KhanDavid ZopfsMohammad JamshidiDavid ZweikerMohammad Mahbubur Rahman Khan MamunDavide BorroniMohammad R. SalmanpourDavide CavagnettoMohammad ShahbakhtiDavide DonnerMohammed DiykhDavide FirinuMohammed R. S. SunoqrotDavide LocatelliMohammed RohaimDavide LonatiMohd Imtiaz NawazDavide LugliettoMohiuddin AhmedDavide MedicaMonica FranzeseDawid PolapMonika GawałkoDazhi JiangMonika KlimekDebarshi RoyMonika KoziełDeborah GalpertMonika Maria BiernatDebra EhrlichMonika SkorupaDechan ChenMonika SobočanDejan JakimovskiMonika SzturmowiczDejan NikolicMonique ReijnierseDelia Mirela TitMootaz SalmanDelia TitMoradi FarshadDenis A. CusackMorag LewisDenis BuxtonMorgan BroggiDenis QuerleuMoritz MeyerDenisa MuraruMoser HeleneDenise HaslwanterMostafa KhairDenise Paschoal SoaresMotoki FukudaDennis VriensMozhgan AlaeddiniDerek PhebyM.S. ZobaerDer-Shin KeMubarak MashrahDesiree Nadine WusslerMufti MahmudDevanshi SethMuhammad Attique KhanDevika GanesamoorthyMuhammad FaisalDevis BenfaremoMuhammad Faizan ShiraziDhamodharan VenugopalMuhammad SoyfooDharam AblashiMukul VijDhaval ChauhanMuqing LiuDhrubajyoti BandyopadhyayMurali MaruthamuthuDhruvajyoti RoyMurtadha HssayeniDiana M. LindquistMusaffe TunaDiana MartinsMustafa S. SalmanDiana Sorina FeierMyra HosmilloDiane AddieMyriam Van WinckelDianpeng QiMyron ChristodoulidesDíaz-Reval IreneN. Facente ShelleyDiego Dos Santos FerreiraNabil EidDiego García-GilNabil KarahDiego RaimondoNadège KindtDietmar FuchsNadim HaboubiDimitri PoddigheNadine PriceDimitrios A. VrachatisNadja EngelDimitrios K FilippiadisNageswara PilliDimitrios KaramanisNagy AkosDimitrios KatsareliasNaijung ChiangDimitrios PilalasNaji KharoufDinesh Babu SomasundaramaNalini ChintalapudiDinesh JillellaNam D. TranDinesh K. ThekkinkattilNanako KawaguchiDingkun RenNancy CasanovaDinh Toi ChuNancy E. KemenyDionicio Angel Galarza-DelgadoNancy SolomonDivya BeriNaoka MurakamiDivya RamchandaniNaoki KawaharaD.M. CopolovicNaoki MorimotoDmitri NepogodievNaoki NakagawaDmitrii S. MaltsevNaoro TanakaDmitry A. MaslovNaosuke KuraokaDmitry BordinNaoya HasegawaDmitry EnikeevNarayanaganesh BalasubramanianDmitry KostyushevNarendiran RajasekaranDmitry V. RoshchupkinNaresh Kumar RavichandranDomenico BaccellieriNaside MangirDomenico DalessandriNasser Said-Al-NaiefDomenico GalettaNatalia D. GladkovaDominika GłąbskaNatalia DłużniewskaDominique J.J. Van NesteNatalia IssaevaDomizia VecchioNatália MartinsDonat AlparNatalia RakislovaDonatella LucchettiNataliia MelnykovaDonato D’AgostinoNatalija PolovicDong Jun OhNataliya RomanyukDong Sik KimNatasa MilosevicDong-Guk PaengNathalie BardinDonghan YangNathan GluckDongheon LeeNathan K. EvansonDong-Oh SeoNaveen KondruDonovan McGrowderNavid SobhaniDoreen SchmidlNeel DesaiDorin HarpazNeeta Upendra KumarDorota A. Darmochwał-KolarzNegar FarzanehDorota WłogaNeil CarletonDörte SchulteNeil McgregorDragana GabrićNektarios KalyvasDragana KomnenovNelly Raymond ZiadéDragana M. StanojevicNemanja JovicicDrago StrleNgee Han LimDragos Catalin JianuNguyen Minh DucDragos CozmaNguyen Quoc Khanh LeDragos MedianNiall McAlindenDrozdstoy StoyanovNian-Sheng TzengDuaa DakhlallahNicholas G. JamesDulskas AudriusNicholas M. KiuliaDya Fita DibweNicholas WeatherleyDylan J. KleinNico HoffmannE. Mark WilliamsNicola IngramE Scott SillsNicola MagnavitaEbbe EldrupNicola MarottaEberhard GrambowNicola MicaleEberhard UhlNicola MontemurroEddy Wai Yeung WongNicola Riccardo PuglieseEdgar Fabián Manrique-HernándezNicola SalvadoriEdmundo Rosales-MayorNicola Stefano FracchiollaEdoardo ErrichielloNicolae CrisanEduardo Collantes EstévezNicolae GicaEduardo Gutiérrez-AbejónNicolas KozakowskiEduardo OliverNicole Noren HootenEdvardas DanilaNicole NovroskiEdward J. PavlikNicoleta AntonEdward R. SauterNicoletta ParucciniEdward TobiasNicolò CardobiEdward ZimbudziNicolo De PretisEdyta BarnasNicolò GirolimettoEero T. HippeläinenNidaa MikaïlEfstathios PettasNigel TempertonEhsanul Hoque ApuNikhil VasdevEihab BedawiNikita MargaryantsEiichi MomotaniNikki ReinemannEiichi WatanabeNikolai Yu. ZolotykhEiji KiyoharaNikolaos A. StavropoulosEiji NanbaNikolaos AntonakopoulosEinar Wilder-SmithNikolaos FragakisElaine ChowNikolaos MachairasEleftherios ChatzellisNikolaos MachairiotisElena BencurovaNikolaos Nikitas GiannakopoulosElena BertelliNikolas K. KnowlesElena CasiraghiNikolas Von BubnhoffElena I. RyabchikovaNikos ViazisElena McBeathNilanjan LodhElena NovákováNils GroBe HokampElena SavvateevaNima SayyadiElena TarcaNiranjan BaluElena V. TchetinaNitin SaksenaElena ZharkikhNoboru IdenoElena-Laura AntohiNoboru OriuchiEleni MavrogonatouNobuhiro AriyoshiEleonora NicolaiNobuhiro HayashiEleonora NuceraNobumichi KobayashiEleonore FröhlichNoel WeissElettra MancusoNoelia Carmona-VicenteElia VecellioNoemí De-los-Santos-ÁlvarezEliane Pedra DiasNooshin AbbasiElias VasiliadisNorbert F. KissElin StorjordNoreen GleesonEline A.J. WillemseNorihiko NaritaEline F. M. OerlemansNorio MiyoshiElio BignardiNoriyuki OhkuraElio F. De PaloNoshir MehtaElisa BaratellaNosotti MarioElisa BelluzziNoura AlsufyaniElisa CairrãoNowicki AndrzejElisa PalazzariNuno S. OsórioElisa ScalcoNuno ValeElisa ZavattaroNurdan TozunElisabeth KapakiNuwan DharmawardanaElisabeth LippertOdongo Steven EyobuElisabetta CostantiniOgnjen BarcotElizabeth A. WhitcombOksana SintsovaEllen Christina ObermannOlaf LarsenEllen Gelpi-MantiusOlayinka A. AiyegoroElmar KotterOleg BlyussEloisa UrrechagaOleg O. MyakininEl-Sayed H. IbrahimOleksii SkorokhodElvira IngleseOl’Ga A. TarasovaElzbieta RadzikowskaOlga AmaralEmanuel K. ManesisOlga BibikovaEmanuela TurillazziOlga Graciela ScharovskyEmanuele MicaglioOlga N. BerdinaEmanuele PivettaOlga SergeevaEmanuele PravatàOlga SisoevaEmilia OgodescuOlga Y. KorolkovaEmilio EstebanOliver SanderEmily JaehneOlivier HuckEmily PorterOllie Yiru YuEmma BrannOluwatosin OluwadareEmma D’IppolitoOmar BouamraEmmanouil N. MagiorkinisOmbretta MelaiuEmmanuel FajardoOmer DeperliogluEmmanuel J. FavaloroOmid Madadi-SanjaniEmmanuel MelloulOndrej BernatikEmyr BenbowOndřej KalitaEndika Prieto-FernándezOndřej NaňkaEne Cosmin- VictorOrazio SchillacoEng Guan ChuaOrnella MilanesiEng Tat AngOsamu YoshinoEnric ReverterOscar Mancera-PáezEnrico BaldiÓscar S. GallegoEnrico GlaabOskar ZakiyanovEnrico MarinelliOsmindo Rodrigues Pires JúniorEnrico MartinOurania TsilomitrouEnrique Gomez GomezOvidiu SirbuEnrique Meléndez-HeviaOya CingozEnzo Maria VingoloP. W. KämmererErberto CarluccioPablo Blanco Martínez De MorentinErdenebayar UrtnasanPablo De GraciaErdenebileg BatbaatarPablo HerreroEren KurisPablo S. RivadeneiraEric ConcholaPablo V. YagupskyEric CrubezyPajor LászlóÉric DaugasPamela Di GiovanniEric SobolewskiPamela TozzoErica Di MartinoPanagiota MikouErica MenegattiPanagiota MitrouErich CosmiPanagiotis PapanagiotouErich MöstlPanagiotis PapoutsoglouErik BehringerPanagiotis PeitsidisErik EdströmPanagiotis TheofilisErmelinda De MeoPanagiotis TsakanikasErnesto Di CesarePaola FeracoErnesto Santiago González MesaPaola GavazzoErshuai ZhangPaola MartinganoEsdras A. ArrietaPaola ParenteEstanis NavarroPaola ParlantiEsteban Obrero-GaitánPaola PasqualiEster Leno-DuránPaola StraticòEsther Diana RossiPaola TombesiEsther F. VicentePaolo AseniEsther Medrano-SánchezPaolo BoffanoEsther Serrano-PertierraPaolo CampioniEtienne RouxPaolo CasadioEtsuko MiyagiPaolo CavorettoEuishin Edmund KimPaolo CrippaEun Hee SohnPaolo Di GiamberardinoEun Jeong GongPaolo FerrariEun-jee OhPaolo ImmovilliEun-Woo LeePaolo MarraEurydiki MichalisPaolo MenèEva AusóPaolo SpinnatoEva GijbelsPaolo TrucilloEva JoverPaolo ZaffinoEva KatonaParaskevas GkolfakisEva KriegovaParasuraman PadmanabhanEva Levring JaghagenParis LaskarisEva M. Medina-RodríguezPasquale CianciEva Mischak WeissingerPasquale Di MaioEvanthia E. TripolitiPatil ShankargoudaEvelise BrizolaPatricia A. BroderickEvelyn Rivera-ToledoPatricia Esmeralda Carreira-DelgadoEvert-Jan P.A. VonkenPatricia MellinEvgenii BelykhPatricia P. WadowskiEvgeny V. SidorovPatricia PriceEwa Osuch-WójcikiewiczPatrick LammieEwa Puszczałowska -LizisPatrick McClureEwa StachowskaPatrick NaughtonEwa TomaszewskaPatrik AnderssonEwa ZiętkiewiczPatrizio MazzoneEwaryst TkaczPatryk LipinskiEwelina DratkiewiczPau Redón LurbeEwert BengtssonPaul A. BlairEzzat KhanPaul A. FuerstF. CarameloPaul BălănescuFabian TroschelPaul BastardFabio CoppedèPaul E. SummersFabio Di NardoPaul Li-Hao HuangFabio GuoloPaul MuhleFabio ManfrediniPaul N. ManleyFabio ZugniPaul SchoenhagenFabrice MeriaudeauPaul SloweyFabrizio AnniballiPaul T. ThevenotFabrizio BertelloniPaula A. OliveiraFabrizio BrontePaula MeleadyFabrizio D’AscenzoPaulina NastałyFadil BidmosPauline HarperFahad QuhalPaulo A. Raymundo-PereiraFaidon-Marios LaskaratosPaulo Alexandre S. Armada Da SilvaFaisal JamilPaulo AlvesFaiza KalamPaulo MotaFangfang TangPaulo R.B. FernandesFarhad MalekiPaulraj KanmaniFarzad Taghizadeh-HesaryPavel CaldaFatemeh KhatamiPavel DundrFatih AbasyanikPavel SukFausta LuiPavol JanegaFawzia Bardag-GorcePavol SuloFederica AmitranoPaweł KalinowskiFederica BellussiPaweł KleczyńskiFederica FinettiPaweł KrzyżekFederica FogacciPaweł LinekFederica IlardiPaweł PomastowskiFederica SabatiniPaweł RussekFederica VernuccioPawel ZmoraFederico FerrariPedro Boal-CarvalhoFederico MeiPedro BrugarolasFederico Paolini PaolettiPedro FerreiraFedor NovikovPedro M. VieiraFei GaoPedro Manuel Moreno-MarcosFeipei LaiPedro Miguel RodriguesFelice SiricoPedro Romero-ArocaFeliciana Real-FernándezPei-Yuan SuFelipe KazmirczakPeng GuoFelix AbererPeng LinFelix ClanchyPengfei ZhangFelix EllettPer AlstergrenFelix M. MottaghyPer StålFeng YuanPernille HolmagerFerenc IzbékiPetar OzretićFerenc PappPetca Razvan-CosminFernando CardosoPeter BrossartFernando De AndrésPeter CelecFernando Henrique MagalhãesPeter DankerlFernando LopezPeter GibbsFernando Manuel Falcáo-ReisPeter HollanderFerreira Nelson FerreiraPeter HowellFerruccio ContePeter Klaus HunfeldFilipe ElvasPeter KokolFilipe PintoPeter KornpratFilippo AucellaPeter KorstenFilippo BaldacciPeter ReismannFilippo CademartiriPeter RumneyFilippo LetoPeter UlriksenFilippo MacalusoPeter Van VeldhuizenFilippo MaselliPeter WhiteFilippo TorielloPetr PohunekFilippos TriposkiadisPetr TvrdikFiliz OezkanPetra HrubáFlaminia VenaPetra KoraćFlemming AndersenPetra ZusterzeelFlorent GobertPetrik GalvosasFlorentina PluteanuPetro E. PetridesFlorian ErgerPetros P. SfikakisFlorian GesslerPhilipp BergFlorian ReckerPhilipp GensekeFlorian RenosiPhilipp MayerFloris Van VeldenPhilipp S. LangeFocke ZiemssenPhilipp TschandlFotios GioulekasPhilippe RobertFrancesc García GonzaloPhilippe SoyerFrancesca AmbrosiPier Francesco FerrucciFrancesca BigazziPier Sergio SabaFrancesca CaumoPiergiorgio La RosaFrancesca LombardiPiero PapiFrancesca PesciniPierre A. MaquetFrancesca SanguedolcePierre VerrelleFrancesca VipianaPietro Andrea BonaffiniFrancesco AsciPietro AuconiFrancesco AulicinoPietro CacialliFrancesco BennardoPietro Di SantoFrancesco BertoliniPietro FusaroliFrancesco BiancoPietro Maria BaveraFrancesco CanonicoPietro SpennatoFrancesco ChianconePing RenFrancesco De CobelliPio ContiFrancesco Del GiudicePiotr AlsterFrancesco Di GennaroPiotr B. HeczkoFrancesco FaitaPiotr DobrowolskiFrancesco FerriniPiotr DuchnowskiFrancesco FortarezzaPiotr KorczynskiFrancesco GallettiPiotr WilczekFrancesco GaziaPipitsa N ValsamakiFrancesco GentilePires IvanFrancesco GigantiPiyush SharmaFrancesco GirolamoPo-Cheng ChenFrancesco MassariPo-Jen HsiaoFrancesco MerollaPouya AlaghbandFrancesco MoraPo-Yu KaoFrancesco PanzutoPrabhudev Prasad PurudappaFrancesco Paolo FanizziPrakash GudsoorkarFrancesco PetrellaPramod RaoFrancesco PlottiPranav SharmaFrancesco RadicoPranshu SahgalFrancesco SalzanoPrashant ChittiboinaFrancesco SanvitoPrateek LohiaFrancesco Saverio SorrentinoPratip BhattacharyaFrancesco SecchiPrativa SahooFrancesco SessaPraveen Ramakrishnan GeethakumariFrancesco SignorelliPreeti ChandraFrancesco SpannellaPriscila GiavedoniFrancesco TovoliPriscilla GuglielmoFrancesco VasuriPritam PandaFrancesco VerdePrzemysław DudaFrancine DurocherPuppo FrancescoFrancis William StaffordP.V. RavindraFrancisca DiasQian DuFrancisca Garcia-MorenoQian HuangFrancisco CarmonaQian ZhangFrancisco Cavas-MartínezQingyu ChenFrancisco EnguitaQiu Jiantai TimothyFrancisco González-ScaranoQu DengFrancisco José González-MineroQuazi RahmanFrancisco MesaQuirina Santos-CostaFranco BassettoR. KocyłowskiFranco LacconeR. Michael Van DamFrançois JamarRabea AslehFrancois MeurensRachael SugarsFrane PaicRachel Bar-ShalomFrank C.T. SmithRachel DaleFrank RoemerRachy AbrahamFranklin Wang-ngai ChowRade DrmanacFranz ZehentmayrRadoica JokićFred PriorRadosław ChaberFrederic Carsten SchmeelRadosław KazimierskiFrederic GrenouilletRadosław KempińskiFrederik HoogwaterRadosław RolaFrickmann HagenRadu ChiceaFulvio BorellaRadu TamaianFulvio RattoRaelene EndersbyFumiaki UchiumiRafael Enríquez De SalamancaFumitaka KogaRafael LlobetFu-Zong WuRafael Lomas-VegaGábor Tamás SzabóRafał Białynicki-BirulaGabriel D. PeckhamRafal DankowskiGabriela-Dumitrita StanciuRafał FilipGabriele CapursoRafal KocylowskiGabriele Di GesaroRafal KrenkeGabriele GhettiRafał MańczakGabriele RondoniRafal MoszynskiGabriele TonniRafał WatrowskiGabriella CasalinoRaffaele NatellaGabriella EmriRaffaele RausoGabriella GuarnieriRaffaella BrugnoniGabriella HorváthRaffaella LombardiGad GiladRaghavendran ParthaGaël PitonRaili MüllerGaetano GalloRaimondo PianaGaetano IsolaRaja VeerapandianGaetano PaoneRajesh SinghGagandeep KaurRajikha RajaGajanan N. SapkalRajinder DawraGalina IlievaRajiv SinglaGalina ShepelkovaRalf KetterGalina V. KurlyandskayaRalph MasonGalliera Emanuela RitaRaluca Simona CostacheGanbayar BatchuluunRam DickmanGannon C. K. MakRam Kumar SelvarajuGarifallia SakellariouRam SamujhGary D. SteinbergRama Sashank MadhurapantulaGary KohanbashRamgopal KashyapGatikrushna PanigrahiRami HallacGaurav D GaihaRami S. KantarGaurav GoelRamon AyonGaurav JoshiRamya Lakshmi RajendranGaurav SharmaRamzi KhamisGavriel ChaushuRana S. HodaGebhard MathisRaquel Leirós-RodríguezGeertruida Hendrika De BockRashad ZayatGelsomina MansuetoRasmus Sejersten RipaGennaro CormioRatheesh Kumar MeleppatGennaro MusiRavi Chand BollineniGennaro VessioRaymond H KimGeorg PongratzRǎzvan PetcaGeorge CarnellRebecca SkalskyGeorge KatselisReem S. Abu-RustumGeorge LazaroiuReema SinghGeorge Mihai NitulescuReinhild PrangeGeorge NotasReinhold FeldmannGeorgi GeorgievRemigiusz BąchorGeorgi LukovRenata GržićGeorgios AndroutsopoulosRenato CuocoloGeorgios BenetosRenato GondarGeorgios D. MichailRene CorteseGeorgios NikolopoulosReza AlizadehGeorgios S MarkopoulosRicardo CoelhoGeorgios SamanidisRicardo ColettaGeorgios VelonakisRicardo RibeiroGeorgios VrakasRicardo VardascaGerald J. KostRiccardo GobbiGerald R. MarxRiccardo La GrassaGerardo PellegrinoRiccardo LattanziGerasimos P. SykiotisRiccardo MasettiGerd MelkusRiccardo MoiaGereon ScharesRiccardo ZojaGergana G. ZahmanovaRichard HolyGerhard C. HildebrandtRichard LemmersGermana De NucciRichard TrohmanGhada ZamzmiRichard WamaiGhais KharmandaRiemer H.J.A. SlartGiacinto BagettaRifat AhmedGiacomo DomeniciRikiya TakeuchiGiacomo MiserocchiRinaldo PellicanoGian BusettoRita ConigliaroGian Paolo CavigliaRita Del PintoGianCarlo PecorariRitsuko Kimata PoohGianfranco MitacchioneRizwanAli NaqviGianluca EspositoRobert AncuceanuGianluca LuccheseRobert Andre GaudinGianluca SerafiniRobert BestGianluca SmerilliRobert ĆelićGianluigi PastaRobert HudecGianni LazzarinRobert J. MorellGianni PesRobert JachGianpaolo CarrafielloRobert KissGiau VoRobert KleszczGiedrius BarauskasRobert LalondeGilbert LimRobert LeMoyneGilles MonifRobert NakayamaGino SeravalleRobert NordstromGionata FragomeniRobert PrentnerGiorgia BeffagnaRobert S BradleyGiorgia CaruanaRobert SifrerGiorgia MelliRobert T. MeansGiorgio BoganiRobert ZymlinskiGiorgio FassinaRoberta GhidoniGiorgio ManginoRoberta MaraglianoGiorgio NovelliRobertas DamaševičiusGiorgio SantoroRoberto CampagnaGiorgio TregliaRoberto CannataroGiovanna FerraioliRoberto CopettiGiovanni BarassiRoberto EspositoGiovanni BenfariRoberto FeliceGiovanni BocciaRoberto NardiniGiovanni CiliaRoberto PiroGiovanni CimminoRobin Singh BhadoriaGiovanni Codacci-PisanelliRobyn Laura KosinskyGiovanni ColonnaRodney HicksGiovanni CrisafulliRodolfo RedaGiovanni FabbriniRodrigo De Paula BaptistaGiovanni Luca RomanoRodrigo Martín San AgustínGiovanni PalleschiRoger BiringerGiovanni PaolinoRoger L. SurGiovanni PellacaniRoger M. KrzyżewskiGiovanni RalliRohan JoshiGiovanni RostiRohan SharmaGiovanni SalzanoRok DolenecGiovanni SuccoRoland HustinxGiovanni TazzioliRoland ZüstGiovanni TossettaRomain BasmaciGiovanni VitaleRomain SebanGiulia AbateRon BardinGiulia BIVONARonald B. BrownGiulia BuizzaRosa María López-Pintor MuñozGiulia Di DalmaziRosalba MansiGiulia FestaRosana Wing-Shan PoonGiulia SiravegnaRosanna Suzuky PintoGiuliano De CarolisRosanna VillaniGiuliano RamadoriRosario CaltabianoGiuliano ZabucchiRosellina MancinaGiulio DiMizioRoss ChelohaGiulio Francesco RomitiRossella BrunoGiulio FrancoliniRossella CacciolaGiuseppe BanfiRossella MieleGiuseppe BarlettaRossella PalmaGiuseppe BertozziRoubini ZakopoulouGiuseppe BroggiRounak DeyGiuseppe Danilo Di StasioRoxana ȘirliGiuseppe FanettiRoy MoncayoGiuseppe FiorentinoRuben Manuel Luciano Colunga BiancatelliGiuseppe G. LoscoccoRuben MedinaGiuseppe IettoRuben PauwelsGiuseppe LanzaRuben UsamentiagaGiuseppe Lucio CasciniRudin PistulliGiuseppe MiserocchiRudolf OehlerGiuseppe MurdacaRune MatthiesenGiuseppe NassoRupsa DattaGiuseppe PernaRute PereiraGiuseppe PontilloRute SantosGiuseppe RasoRutger J. Van BommelGiuseppe RicciRuth KaufmannGiuseppe RivaRuth ParksGiuseppe SantarpinoRuxandra JurcutGiuseppe SimoneRuxandra MareGiuseppe TroianoRwik SenGiuseppe VarvaraRyad ZemouriGiuseppe VetrugnoRymantė GleiznienėGiuseppina E. GriecoRyogo MinamimotoGiuseppina RizzoRyong Nam KimGiuseppina T. RussoRyota ShizuGladys HoRyuichi OhtaGlenn BaumanRyuji HamamotoGloria Álvarez-SolaSa Ra LeeGloria PascualSaba TabasumGonzalo VarelaŞaban ÖztürkGopi BattineniSabbir KhanGoran CuricSabina SevcikovaGordana IvanacSabine ZangeGordana Wozniak-KnoppSaburo MatsubaraGrady NelsonSaeed JerbanGraziella Di GreziaSaima MushtaqGraziella TuratoSajjad ShiraziGrazyna Kaminska-WinciorekSalah A. MohamedGregorio Bernabe GarciaSalvatore Massimiliano CardaliGregorio CordónSalvatore SacconeGregory KoronakosSalvatore ScaccoGriet L.R.L. GlorieuxSameh AbdelnourGrigorios KorosoglouSameh GehaGrzegorz ŁabuzSami AyramoGualtiero AlvisiSami P. VäänänenGuang YangSamir BarghoutGuangai XueSampurna ChatterjeeGuangdi LiSamuel ClokieGuangyao WuSamuel NavarroGuanqiu QiSandeep KumarGuennadi SaikoSandeep Kumar MishraGuglielmo CampusSandeep Kumar VashistGuglielmo ManticaSandeep SinghGuido Alberto Massimo TiberioSandeep VashistGuido GiordanoSandi Baressi ŠegotaGuigen LiuSandi KweeGuillaume DucarmeSandip N. ChavanGuillaume LandrySandipto SarkarGuillaume LefebvreSandor BenyheGuillermo Pérez-PërezSandra LejnieceGuillermo PlazaSandra P. NunesGülen YerlikayaSandra Rovira-AmigoGulisa TurashviliSandra StoppelkampGulzhanat AimagambetovaSandrine BourgoinGun Oh ChongSandrine ChamayouGunjan AroraSandy EldridgeGunnar SchuetzSang Hyun ChoiGunnevi SundelinSang Tae ChoiGüntülü AKSangah HanGuodong ZengSang-Yeub LeeGuorong LiSanja Popovic-GrleGusztav BeltekiSanjay PhogatGuy R. AdamiSanjiv SharmaGuy RostokerSanthosh MukundanGwen M. ClarkeSantiago MelónGyanendra P. JoshiSantos MafaldaHaekyung Jeon-SlaughterSantosh GeorgeHae-Seong NamSantosh PandaHaewon ByeonSara CostanzoHafiz Tayyab RaufSara IserniaHaipeng LiuSara MocciaHamid Reza MaratebSara Monteiro-ReisHamza El HadiSara Ortiz-ToqueroHan Naung TunSara ShimoniHan ZhangSarah L. SawyerHania Salah ZayedSarath VijayakumarHankyu LeeSarel HalachmiHanna BandarenkaSarhang Sarwat Hama GulHanna GerberSarita PrabhakaranHanno M. WitteSarmila TandukarHans BockSarv PriyaHans-Christian SiebertSascha TreskatschHans-Jonas MeyerSashi KantHans-Peter SimmenSatadru LahiriHaoran WeiSatish Balasaheb NimseHarish PatelSatoshi EbataHartmuth NowakSatoshi NishidaHaruki KoikeSatoshi OedaHarun IlhanSatoshi SakaiHarvinder Singh ChhabraSatoshi TakahashiHasan AbedSatu HepojokiHassan KassassirSatyabrata AichHazizul Hussain-YusufSatyavani KaliamurthiHector FerralSaulius CicènasHee Chan KimSaulius LukoseviciusHee Yoon JeongSaulius LukoševičiusHee-Cheol KimSaulius RočkaHeidi SowterSaulius RutkauskasHeiko TzschatzschSavvas LampridisHeiner Von ButtlarScott ReidHeinrich GerdingSean Chun Chang ChenHeinz ZollerSean P.F. HughesHei-sung KimSeason S. WongHeldring NinaSebastiaan TheunsHélène CastelSebastian BürkleinHélène Niculita-HirzelSebastian D. SchaeferHelge Egil Seime PettersenSebastian KrugHelios De RosarioSebastian M. JudHelmet T KarimSebastian MajewskiHelmut H. PopperSebastian TancoHemant K. MishraSebastian TschaunerHenk KoppelaarSebastiano CiccoHenning ReisSebastjan BevcHenrique SilvaSeiichi KitagawaHenry Dushan E. AtkinsonSeiichiro AbeHenry SutantoSeila Lorenzo-HerreroHervé ReychlerSelene BaroneHerwig CerwenkaSenka MeštrovićHesam BabahosseiniSenol PiskinHestia S. MellertSeong Hee AhnHidefumi KohSeongjae KimHidehiko OkazawaSerafeim MoustakidisHideki FujiiSerafin MoralesHideki MiyachiŞerban Vifor Gabriel BerteşteanuHideki SekiyaSerena MilanoHidenori SuganoSergei NekhaiHideo KatoSergey KornilovHidetoshi KomatsuSergey KozlovHideyuki ChibaSergey MoskvinHimanshu BatraSerghei CebotariHimanshu GuptaSergio A. UsecheHimanshu KathuriaSergio CurtoHinnerk Schulz-HildebrandtSergio D’AddatoHiraku SasakiSergio De SalvatoreHiroaki KonishiSergio HaimovichHiroaki NagashimaSeung Bae HwangHiroaki NakagawaSeung Sam PaikHirofumi FujiiSeung-ho LeeHiroki TeragawaSeung-Hun LeeHiromi KurokawaSevastita IordacheHironori IshiguchiSeverin RodlerHiroshi FujishimaSeyedmehdi Mehdi D. PayabvashHiroshi KurokiShahanshah KhanHirotsugu FukudaShahin OftadehHirotsugu MaruyamaShamik ShahHiroyoshi MoriShamus KeelerHiroyuki NakanishiShang-Ming ZhouHisahide NishioShang-Yu WangHisashi ShigaShankar Jaikishan EvaniHisato YagiShanshan TanHo Sun ShonShaoqiu HeHo Yeon KimShazid Md. SharkerHolger WittigShebli AtrashHong LiangSheeba Jenifer SujitHong PanSheeba SujitHong-Gam LeSheng-Dean LuoHongkai ZhuSheng-Hung ChenHongming ShanShengwen Calvin LiHongtian ChenSheraz GulHongyan DaiShervin MinaeeHongying PanShibin ChengHoon KimShicheng ChenHorea FeierShigeki MatsubaraHoria HaragusShigeo MuroHoria StanescuShigeru NakaiHossein TaghizadehShih-Jen LiuHsieh Bao-YuShih-Yen LoHsin-Yao WangShikha SainiHsu-Heng YenShikha ShrivastavaHuapeng FengShin NishioHuazheng LiangShingo KatoHuet SarahShinichi SuzukiHui HuangShinji KawabataHui-Ching ChuangShinji OkaniwaHui-Hua HsiaoShintaro FujiharaHung ChangShintaro SukegawaHung-Chi ChenShipra Agarwal AgarwalHung-Vu TranShogo ShinoharaHurriyet YilmazShoko KureHuub H. Van RossumShruti ChandraHwa-jung KimShugo MizunoHyeck-Soo SonShuhei NakaHye-jin ShimShuhei YamadaHyun Jin KwunShuji OzakiHyun Jun ParkShuo ZhangHyung Jun ParkShu-Yan LiHyun-Jae ChoShyunji HayashiIacopo PetriniSiamak DoostIan M. ThornellSiddharth DugarIbrahem KandelSied KebirIbrahim M. SayedSiew-Kee LowIdris RajiSi-Hyun KimIgnacio Ruz-CaracuelSilia VitoratouIgnazio Gaspare VetranoSilvia De Miguel-BilbaoIgor DumicSilvia M. Sanz-GonzálezIgor LebedevSilvia MascoloI-Hsiu HuangSilvia Rocchiccioli‪Ikechukwu AchilonuSilvia SpivakovskyIkuya MiyamotoSilvia TerraneoIlaria IacobucciSilvia TommasinIlaria StadiottiSimon BaileyIlia FishbeinSimon HeekeIlias ElmoukiSimon PodnarIlias SachpazidisSimon SchröderIlkka IlonenSimon ScottIlko IlevSimon W. RabkinIlona KopytaSimona BernardiIlona KovalszkySimona CimaIlona RybińskaSimona CristescuIl-Youp KwakSimona GeorgievaImdadur RahmanSimona MartinottiImmonen MillaSimona MoldovanuImran FarooqSimona NicoaraIn Seok MoonSimona SperlonganoIneke Van Der BurgtSimone BelmontiInês SilvaSimone GrassiIngemar FredrikssonSimone MorselliInna S. MidzyanovskayaSimone SchüllerInnokentii E. VishnyakovSimone SerafiniIoan TileaSimone SforzaIoannis A. GiantsisSimone TambaroIoannis IakovouSissel Kronborg-WhiteIoannis SamarasSiu Wai ChoiIonut SchiopuSiva Krishna AngalakurthiIrena JekovaSivabaskari PasupathyIrena TabainSivaraman NatarajanIrene Hernández-GirónSlawomir TeperIrene Lo CignoSmaragdi MarinakiIrene MadrigalSnježana Mardešić-BrakusIrina BonzheimSobuj MiaIrina SominskayaSofia PessanhaIryna SamarskaSofia RavaraIsaac RuizSofija SpasojevićIsabel LarreSoichiro MurataIsabel LegazSomashekar G. KrishnaIsabel MurSon Long HoIsabel PinillaSonam KumariIsabella CastellanoSonia D’ArrigoIsabella GaragiolaSonia Helena Contreras-OrtizIsam BsisuSonia MorettiIsao IshiiSonja GamsjaegerIsao OtsukaSoon-Chan KwonIsidro García-MeniñoSoonchul LeeIsmael BuñoSophie I. MavrogeniIsmail ZaedSophie MavrogeniIsmini E. PapageorgiouSophie RichterIulia AndrasSøren Baarsgaard HansenIulia-Cristina BagiuSotiris KotsiantisIva KiracSougata GhoshIvan BratchenkoSourav PatnaikIvan DunđerSourish GhoshIvan S. KiselevSousa David CordeiroIvan StefanovicSousana PapadopoulouIvan Y. IourovSpyridon KanellakisIvana KholováSrinivas AluriIvana MaidaSrinivas NellimarlaIvana SamarzijaStanescu Ana Maria AlexandraIvana ŠkrlecStanisław NiemczykIvanka DimovaStavros J. BaloyannisIvo Dumić ČuleStefan Cristian VesaIvo GuberStefan D. MomčilovićIvo P. Torres FilhoStefan HohausIzabela RaganStefan J. LangIzabella Karska-BastaStefan LohseJ. Michael SorrellStefan MalafaJaber AlqahtaniStefan MihaicutaJacek CapalaStefan PittnerJacek GębickiStefan SajdakJacek SiódmiakStefania GalimbertiJacek SzymańskiStefania MarsiliJack MerrinStefania RizzoJae Yong KimStefano CiardulloJae-Bum JunStefano DastoliJae-Joon HwangStefano D’ErricoJae-Yeol JooStefano Francesco CrinòJaeyul LeeStefano LuzzagoJai Young ChoStefano Marco Paolo RossiJaime Arellanes-RobledoStefano PalermiJaime DevineStefano PalmucciJaime Lora-TamayoStefano RestainoJainil ShahStefano TozzaJakob ThannesbergerStefano ZiccardiJakub DobruchStefanos RoumeliotisJames Bowen StiehlStephan LindemannJames G. RavinStephane ChauvieJames HurleyStephanie CohenJames KuoStephanie DuguezJames LeighStephen CrookeJames V. AquavellaStephen J RichardsJames W. GrauStephen StrumJames W. MacKaySteven BaumruckerJan BorggrefeSteven KnapperJan EggerSteven M. PascalJan HomolakStuart A. McIntoshJan Irma PoelaertStuart HogarthJan Krzysztof NowakSubbaramiah SridharJan KühnischSubrata DebJan KvasnickaSudi PatelJan Martin Sommerlath SohnsSuea-Ngam AkkapolJan PithaSuganthaPriya ElayapillaiJan StrnadelSuhwan LeeJan Van SchaikSukhvinder SinghJan Willem KallewaardSukrit GuptaJan ZabrzyńskiSundeep SinghJan ZauchaSuneel KumarJana NekolováSung Ki LeeJana RieggerSung Noh HongJanarthanan KrishnamoorthySung-Hyoun ChoJanett FischerSungmin KimJanine GronewoldSunil JaimanJanja TrcekSun-Young KongJanna G. KoppeSüreyya SavaşanJ.A.P.M. De LaatSusana Gómez-BarreroJared BarrottSusana Nunes SilvaJaromir AstlSusanna GuerriniJarosław FabiśSushil DubeyJarosław Jaszczur-NowickiSusumu SatoJasenka Gajdoš KljusurićSven FlemmingJasenka WagnerSvetlana TamkovichJasmina Casals TerreSwati JainJasna Lenicek KrlezaSyed Haris OmarJasna MetovicSyed M.B. AsdaqJason KarchSylwia ChojnowskaJason Yongsheng ChanSylwia Dzięgielewska-GęsiakJaume Coll-FontSytske Anne BergstraJavad AlizargarSzymon SkoczyńskiJavier Gonzalez-PenasTae Keun YooJavier Molina-CerrilloTaishi HataJaviera Martinez GutierrezTakaaki SenbonmatsuJay HanasTakahiro AsamiJay S. MishraTakahito NakajimaJaydeep BhatTakashi MatsusakiJean AmiralTakashi MatsushimaJean PastréTakashi NakahariJean VanderpasTakashi OhtsukaJean-Baptiste PinaquyTakaya SasakiJean-Claude SadikTakayoshi ShinyaJean-Francois Lauzon-JosetTakayuki TakedeJean-Francois RualTakehito HananouchiJean-Jacques MonsuezTakemichi FukasawaJean-Philippe BerteauTakeshi AnnouraJean-San ChiaTaku KouroJee Young KimTamas HaideggerJee-Young ParkȚarălungă Dragoș DanielJeffrey HubbardTatsuo KandaJeffrey KnaufTatsuya NakachiJeffrey ThompsonThomas CzypionkaJelena KnezevicThomas SimonJenni KarttunenTiberiu-Paul NeaguJennifer D. Foulke-AbelTilman EmrichJenny QueTing-Wen ChenJenq-Shyong ChanTing-Yi LinJens André HammerlTomasz BlicharskiJens Christian MöllerTomasz KluzJens KleesiekTomasz RakowskiJens KrethTommaso BacciJeong Won LeeTomoaki MiyagawaJeovanis GilTomofumi MisakaJeremy AugustinTomoki NaoeJermaine I. G. CowardTomomari KinoshitaJeroen NieuwlandToshiharu MatsumotoJeroen Van Der HeijdenToshiro HaraJerónimo GonzálezToshiyuki SetoJerzy MackiewiczToyohiro HamaguchiJerzy NarlochTriantafyllos DidangelosJerzy ŚwiątekTrifan Anca-VictoritaJesper Møller JensenTsai-Ching HsuJessica CoertseTsung-Hsien ChangJesus Canora LebratoTsung-I HsuJesús Cosín-RogerTudosa IoanJi Hyun ParkTunde CsepanyJi Won MinTzu-Chieh ChouJialing ZhangTzu-Chuan HoJian Q. YuTzu-Yi ChuangJian ZhouUlrich Friedrich WellnerJianwei ShuaiUmberto CaterinoJibran Sualeh MuhammadV Koneti RaoJie GaoVaitsa GiannouliJie LiValerij Gennadevic KiselevJie XuVaradan SevilimeduJiechao GaoVasileios SiokasJifke VeenlandVera FaustinoJijun HuangVesna ProkicJill P. SmithVictor GueutinJill TippingVijay RamaniJin Wook YiViktor DreminJingfei LiuVincenza GianfrediJingjing TangVincenzo ScuderiJingquan WangVinoth Kumar BojanJin-Hwa JungVioleta MelinteJinn-Rung KuoVirginia D. SteenJinny TaveeVittorio AprileJin-woo KimVladimir JurisicJinyang ZhangVratislav FabianJin-young KimVucenik IvanaJiri HrdyW. D. GrimmJitka ForstovaWahyu CaesarendraJi-Youn SungWei-Chieh LeeJo KatoWei-Jhong JuJoachim HohmannWeijin GuoJoachim KroisWen-Chin HuangJoan VicianoWenhao ShenJoana RoloWenping GongJoanna CabajWilliam R. RodríguezJoanna DepciuchWon Jae KimJoanna Domagala-KulawikWon-jun JiJoanna KonopińskaWoo June ChoiJoanna Petryka-MazurkiewiczWuh-Liang HwuJoanna StefanowiczXiang LiJoanna StragierowiczXiangping ChuJoão Gaspar-MarquesXiaodong GeJoão Mário MirandaXiaofeng Allen SuJoão Miguel Marques Dos SantosXiaohu HuangJoão Pinto-De-SousaXiaowei LiuJoao R GomesXiaoyan ChenJoaquim ChalerXin CuiJoaquin Gayoso CabadaXuezhi JiangJoaquin Manzo-MerinoYana J. AnfinogenovaJochen HillerYasuhito IshigakiJochen MaulYeou-Jiunn ChenJochen WeigtYi-Chou HouJoe JamesYichuan LiuJoel RovnakYi-Hsuan HsiaoJohan H. C. ReiberYing-Chun ChienJohan LindqvistYohei IkezumiJohann SellnerYoko OzawaJohanna FraserYong Qin KohJohannes GratzYonggang PeiJohannes RingYoon-Kyung ChangJohn BennettYoshifumi KasugaJohn BischofYoshifumi SaijoJohn David PrologoYoshihiro FukumotoJohn EakinsYoshihito ShimaJohn ElefteriadesYoshinori ArisakaJohn GriniatsosYoshinori OkamotoJohn Healy J.Yoshiro HoraiJohn HortonYoung-Seob JeongJohn K. YueYuan-Hsi WangJohn L. CliffordYuan-Ti LeeJohn L. ReaganYu-Chan ChaoJohn Mac SharryYu-Chih LoJohn Paul MirozYuhsi HsiehJohn R. BuscombeYuichi MineJohn S TamYuichi RikuJohn T. BatesYuichi SaitoJolanta RzymowskaYuji HirowatariJolanta VaškelytėYuki NakamuraJolanta-Justina VaskelyteYukimasa HatachiJon GaniYuliya I. RaginoJonas KolbenschlagYusen HeJonathan GelfondZehuan LiaoJonathan HyettZhi ChaoJonathan NoujaimZhifeng DuJonathan PanZoard T. KrasznaiJonathan SoZohaib MushtaqJonathan TurnerZoran KarlovićJong Hyo KimZygmunt WarzechaJong Jin Hyun



